# Unsupervised multimodal deep learning for galaxy morphology taxonomy: integrating ConvNeXtEmbeddings and morphological parameters for scalable survey science

**DOI:** 10.1038/s41598-026-45369-5

**Published:** 2026-04-11

**Authors:** I. M. Selim, Ahmed S. Farahat, Lobna H. Basmsm, Afaf M. Abd El-Hameed

**Affiliations:** 1 Faculty of Computer & Artificial Intelligence, University of Sadat City, Sadat City, Egypt; 2 Space Navigation Department, Faculty of Navigation Science and Space Technology (NSST), Beni-Suef University, Beni-Suef, Egypt; 3 Space Environment Department, Faculty of Basic and Applied Science, Egypt-Japan University of Science and Technology, New Borg El Arab, Egypt; 4 National Research Institute of Astronomy and Geophysics (NRIAG), Cairo, 11421 Egypt

**Keywords:** Galaxy morphology, Unsupervised learning, Deep learning, Multimodal fusion, Clustering algorithms, Astronomical surveys, Computational biology and bioinformatics, Mathematics and computing

## Abstract

The rapid growth of astronomical imaging data from next-generation surveys necessitates automated and scalable approaches to galaxy morphology classification that transcend the limitations of supervised methods requiring manual labels. We present an unsupervised multimodal deep learning framework that integrates ConvNeXt-derived visual embeddings with quantitative morphological parameters including concentration, asymmetry, smoothness, Gini, and M20 to uncover natural taxonomic structures within galaxy populations. Using a strictly purged and physically verified sample of 4950 galaxies from the Sloan Digital Sky Survey, we engineered a PyTorch-based Multimodal Autoencoder (MAE) to compress features into a dense 64-dimensional bottleneck, successfully resolving the inherent dimensionality imbalance. Clustering was executed exclusively within this robust latent space utilizing a probabilistic Gaussian Mixture Model (GMM). An explicit ablation study confirmed that this multimodal architecture optimizes structural cohesion compared to isolated modalities. Furthermore, we established the astrophysical integrity of the unsupervised clusters through a new proxy external validation against classical heuristic constraints, achieving a 52.7% baseline alignment. By utilizing GMM log-likelihoods, we isolated extreme physical anomalies (limiting the noise fraction to 2.0%), producing a physically coherent taxonomy that maps seamlessly to Early-Type, Late-Type, and Interacting systems. Each galaxy was processed in ~ 27.6 ms, demonstrating strong scalability for upcoming large-scale surveys such as LSST and Roman. This study establishes a foundation for unsupervised morphology analysis at survey scale, advancing our understanding of galaxy evolution through multimodal deep representation learning.

## Introduction

The visual classification of galaxies based on their morphological structure has been a fundamental practice in astronomy since the early twentieth century^1^. The arrangement of stars, gas, and dust into structures such as spiral arms, bars, and bulges provides a direct window into a galaxy’s dynamical history, star formation activity, and evolutionary state^2^. The foundational Hubble sequence and its extensions by de Vaucouleurs have served as the primary language for describing the observed diversity of galaxy forms, linking morphology to underlying physical properties^3^. Understanding this diversity is crucial for developing and constraining theories of galaxy formation and evolution, from the initial collapse of protogalactic clouds to the complex interactions and mergers that shape galaxies over cosmic time^4^.Modern astronomical surveys, such as the Sloan Digital Sky Survey (SDSS)^5^, the Dark Energy Survey (DES)^6^, and the upcoming Vera C. Rubin Observatory’s Legacy Survey of Space and Time (LSST)^7^, are generating petabytes of imaging data, capturing billions of galaxies. The sheer volume and complexity of these datasets render traditional methods of visual classification by human experts unsustainable. While citizen science projects like Galaxy Zoo have successfully harnessed the collective power of volunteers to classify millions of galaxies^8^, they cannot keep pace with the exponential growth of astronomical data. This data deluge creates a pressing need for automated, scalable, and reliable methods for morphological classification.

In recent years, deep learning, particularly Convolutional Neural Networks (CNNs), has emerged as a transformative technology for image analysis tasks across numerous scientific domains^9^. In astronomy, CNNs have demonstrated high efficacy in supervised galaxy classification tasks when large labeled datasets are availablein classifying galaxies when large labeled datasets are available for training^10,11^. However, creating these large, high-quality labeled datasets is a significant bottleneck. Furthermore, supervised models are often limited to predefined classes and may struggle to identify new or rare morphological types. To overcome the limitations of supervised learning, this work explores the power of unsupervised clustering combined with transfer learning. Transfer learning allows us to leverage the powerful feature extraction capabilities of CNNs that have been pre-trained on massive, general-purpose image datasets like ImageNet^12^. By applying these models to galaxy images, we can extract rich, hierarchical feature representations without the need for galaxy-specific labels^13,14^. These features can then be used in an unsupervised setting to discover the intrinsic morphological groupings present in the data.

This paper presents an innovative hybrid methodology that combines the strengths of two distinct but complementary unsupervised learning approaches. We introduce a hierarchical, multi-scale clustering framework that integrates deep features from multiple CNN architectures with traditional, physically-motivated morphological parameters (e.g. concentration, asymmetry, smoothness)^15^. Our contributions are threefold:

1. A Multimodal Feature Fusion Strategy: We demonstrate that combining deep visual features from both EfficientNet-B0 and ConvNeXt-Base architectures with classical morphological indices creates a more comprehensive feature space for classification.
2. A Hierarchical Multi-Scale Clustering Approach: We propose a two-tiered clustering process that first uses a robust partitional algorithm (K-Means) to identify broad, coarse-grained morphological families and then employs a density-based algorithm (HDBSCAN) to uncover finer, more nuanced sub-structures within each family.
3. A Comprehensive Comparative Analysis**:** We conduct a rigorous evaluation of multiple clustering algorithms (K-Means, Gaussian Mixture Models, Spectral Clustering, and HDBSCAN) and validate our hierarchical approach using a suite of internal validation metrics and statistical tests.

By integrating these advanced techniques, our framework provides a powerful and interpretable tool for exploring the complex landscape of galaxy morphology. This data-driven approach not only recovers the well-established morphological classes but also has the potential to reveal previously unknown patterns of galaxy structure, offering a scalable solution for the scientific exploitation of next-generation astronomical surveys.

## Related work

The classification of galaxy morphologies has a rich history, evolving from qualitative visual schemes to quantitative, automated techniques. This evolution has been driven by the dual needs of imposing order on the apparent chaos of galaxy forms and of linking morphology to the underlying physics of galaxy formation and evolution.

### Morphological classification systems

The first systematic and widely adopted classification scheme was introduced by Edwin Hubble in 1926 and later refined in his 1936 work, *The Realm of the Nebulae*^1^. The Hubble sequence, or “tuning fork” diagram, organizes galaxies into three primary classes: ellipticals (E), spirals (S), and irregulars (Irr). Ellipticals are sub-classified based on their apparent ellipticity, from E0 (circular) to E7 (highly elongated). Spirals are divided into normal spirals (Sa, Sb, Sc) and barred spirals (SBa, SBb, SBc) based on the tightness of their spiral arms and the prominence of their central bulge. This morphological sequence was initially thought to be an evolutionary one, but is now understood to primarily reflect variations in initial conditions, angular momentum, and subsequent merger history^3^.

Gerard de Vaucouleurs later extended the Hubble system into a more detailed, three-dimensional classification volume, often referred to as the revised Hubble-Sandage system^16^. This system introduces intermediate spiral types (e.g. SAB for weakly barred spirals), adds a “family” classification based on the presence of inner and outer rings, and extends the sequence to later types, including Magellanic irregulars (Im). The de Vaucouleurs system provides a more continuous and nuanced description of galaxy structure, reflecting the complex and often overlapping nature of morphological features^17^. While these systems have been invaluable, their application relies on subjective visual assessment, which is difficult to scale to the vast datasets of modern surveys.

### Quantitative morphological parameters

The classification of galaxy morphologies originated with the foundational Hubble sequence and its de Vaucouleurs extensions, which organized galaxies into ellipticals, spirals, and irregulars based on visual characteristics^1,3,16^. While historically invaluable for linking morphology to galactic evolution, subjective visual classification cannot scale to modern survey volumes. Consequently, automated non-parametric indicators were developed. The most prominent are the CAS parameters (Concentration, Asymmetry, Smoothness)^15^, which quantify light distribution, interaction signatures, and star formation clumpiness. Complementary to CAS, the Gini coefficient (G) and M20^18–20^ measure light inequality and the spatial distribution of the brightest regions, robustly separating normal from merging systems. These quantitative parameters provide an objective, albeit low-dimensional, basis for morphological classification and serve as the physical constraints in our multimodal architecture.

### Machine learning in galaxy morphology

The application of machine learning to galaxy morphology has largely followed two paths: supervised and unsupervised learning. Supervised methods, particularly deep learning with CNNs, have achieved impressive results by training on large, labeled datasets from projects like Galaxy Zoo^10,11,21^. These models can learn complex, hierarchical features directly from pixel data, often outperforming methods based on traditional morphological parameters. Transfer learning has proven to be a particularly effective strategy, where a CNN pre-trained on a large, non-astronomical dataset like ImageNet is fine-tuned for galaxy classification^13,14,22^. This approach mitigates the need for extremely large labeled astronomical datasets and has become a standard practice in the field. However, the reliance on labeled data is a fundamental limitation of supervised learning. Unsupervised learning methods, by contrast, aim to discover the intrinsic structure of the data without predefined labels. This is particularly valuable for exploratory analysis of large surveys, where innovative galaxy types may be present. Early applications of unsupervised learning used algorithms like K-Means or Self-Organizing Maps on catalogs of morphological parameters^23^.

Recent advancements have increasingly focused on unsupervised and multimodal deep-learning approaches to extract morphological taxonomies without human bias^24,25^. For instance, Kolesnikov et al.^26^ demonstrated the strong efficacy of self-supervised learning techniques for robust galaxy clustering. Fang et al.^27^ highlighted the potential of CNN-only unsupervised frameworks to discover structural groupings directly from survey images. Furthermore, Ref. 28 have shown that structural features (e.g. spiral arm pitch angles) are deeply tied to local environments, underscoring the necessity of capturing both deep textural features and classical physical parameters to fully map morphological diversity.

The present work builds upon these modern advances by addressing a critical algorithmic gap in multimodal fusion: the severe dimensionality imbalance between vast deep embeddings (e.g. 1000 + dimensions) and scalar physical parameters. Previous unsupervised approaches often struggle with the continuous topology of the Hubble sequence. Rigid algorithms like K-Means improperly force galaxies into artificial spherical boundaries, while density-based methods like HDBSCAN frequently fail on continuous manifolds, rejecting unacceptably large fractions of transitional galaxies as ‘noise’^29^. To mathematically resolve this, we propose a physically-grounded architecture: a Multimodal Autoencoder (MAE) that compresses both deep CNN embeddings and strict morphological parameters into a unified, balanced 64-dimensional latent bottleneck. Within this stable manifold, we employ a probabilistic Gaussian Mixture Model (GMM). Unlike HDBSCAN, the GMM naturally models the continuous probabilistic transitions of galaxy evolution, allowing us to explicitly isolate true physical anomalies using log-likelihood thresholds rather than discarding valid data. This approach represents a mathematically rigorous step forward in the unsupervised extraction of survey-scale galaxy taxonomies.

## Dataset and preprocessing

The foundation of this study is a comprehensive dataset of galaxy images drawn from the Sloan Digital Sky Survey (SDSS) Data Release 17, a major multi-spectral imaging and spectroscopic redshift survey that has mapped a large fraction of the sky^5^. Initially, our sample consisted of a randomly scraped subset of 5000 galaxy images. These are explicitly selected to serve as a scalable proof-of-concept for unsupervised taxonomy extraction in the local universe (z < 0.1). Following the rigorous physical artifact purging protocol detailed in Sect. “Image preprocessing”, our final pristine dataset comprises 4950 galaxies. Each image is a 256 × 256 pixel color composite created from the g, r, and i bands, providing a standardized visual representation for morphological analysis.

### Image preprocessing

To ensure consistency and remove instrumental artifacts, all images underwent a standardized preprocessing pipeline. This pipeline is crucial for homogenizing the data and preparing it for feature extraction. The key steps included background noise suppression and intensity normalization. First, a Gaussian filter with a standard deviation (σ) of 1.5 pixels was applied to each image to smooth out background noise and minor detector artifacts. Subsequently, each image was normalized using a Z-score transformation, which rescales pixel intensities to have a mean of zero and a standard deviation of one. This step is critical for ensuring that the deep learning models are not biased by variations in image brightness or contrast. The baseline pixel transformation is formally defined as:

1 $$f\_norm\,\, = ((I*\,g\_\sigma )\, - \mu \_I)/\,\sigma \_I,$$

where $I*\,g\_\sigma$ denotes the convolution of the original image array with a 2D Gaussian kernel (σ = 1.5) to suppress high-frequency detector noise, $\mu \_I$ and $\sigma \_I$ represent the global mean and standard deviation of the pixel intensities.

Data Purging and Artifact Rejection: To directly address potential segmentation failures and optical artifacts inherent in automated survey pipelines, we implemented a rigorous physical purging protocol prior to deep feature extraction. Images exhibiting mathematically invalid morphological bounds (e.g. C ≤ 1.0, C > 7.0, or A > 1.0) indicating corrupted segmentations were explicitly dropped. Furthermore, an unsupervised Isolation Forest was applied across the morphological parameter space to statistically detect and prune extreme multivariate outliers. This quality-control step successfully isolated and removed 50 corrupted samples, yielding a highly verified, pristine working dataset of 4,950 galaxies.

### Morphological parameter extraction

In addition to the raw pixel data, we extracted a set of five well-established, physically-motivated morphological parameters for each galaxy. These parameters provide a quantitative, low-dimensional summary of galaxy structure and serve as a valuable complement to the high-dimensional features learned by the deep learning models. The extracted parameters are:

- *Concentration* (*C*): A measure of how centrally concentrated the galaxy’s light is, typically defined as the ratio of the radii containing 80% and 20% of the total flux.
- *Asymmetry* (*A*): Quantifies the degree of rotational asymmetry by comparing the galaxy image to a 180-degree rotated version of itself.
- *Smoothness* (*S*): Measures the clumpiness or high-frequency structure in the galaxy’s light distribution, often associated with star-forming regions.
- *Gini Coefficient* (*G*): An index of inequality that measures how evenly the light is distributed among the galaxy’s pixels.
- *M_20 Moment*: The second-order moment of the brightest 20% of the galaxy’s flux, which is sensitive to the presence of bright, off-center clumps like spiral arms or merging nuclei.

These five parameters were calculated for each galaxy following the foundational mathematical formulations established by Ref. 15 for the CAS system, and Ref. 19 for the indices Gini and $M\_20$. Together, they form a quantitative, physical feature vector, $f\__{morph}$, often used in conjunction with other parameters to create a 5**-**dimensional feature space—sometimes referred to as $R^{5}$, ($f\__{morph} \, \in \,\,R^{5}$). Rather than undergoing standard concatenation, this vector serves as the critical astrophysical constraint injected directly into our Multimodal Autoencoder (MAE) bottleneck, discussed in Sect. “Multimodal autoencoder (MAE) and dimensionality balancing”, ensuring that the deep visual features are robustly anchored to established physical realities.

## Methodology

**Fig. 1:**
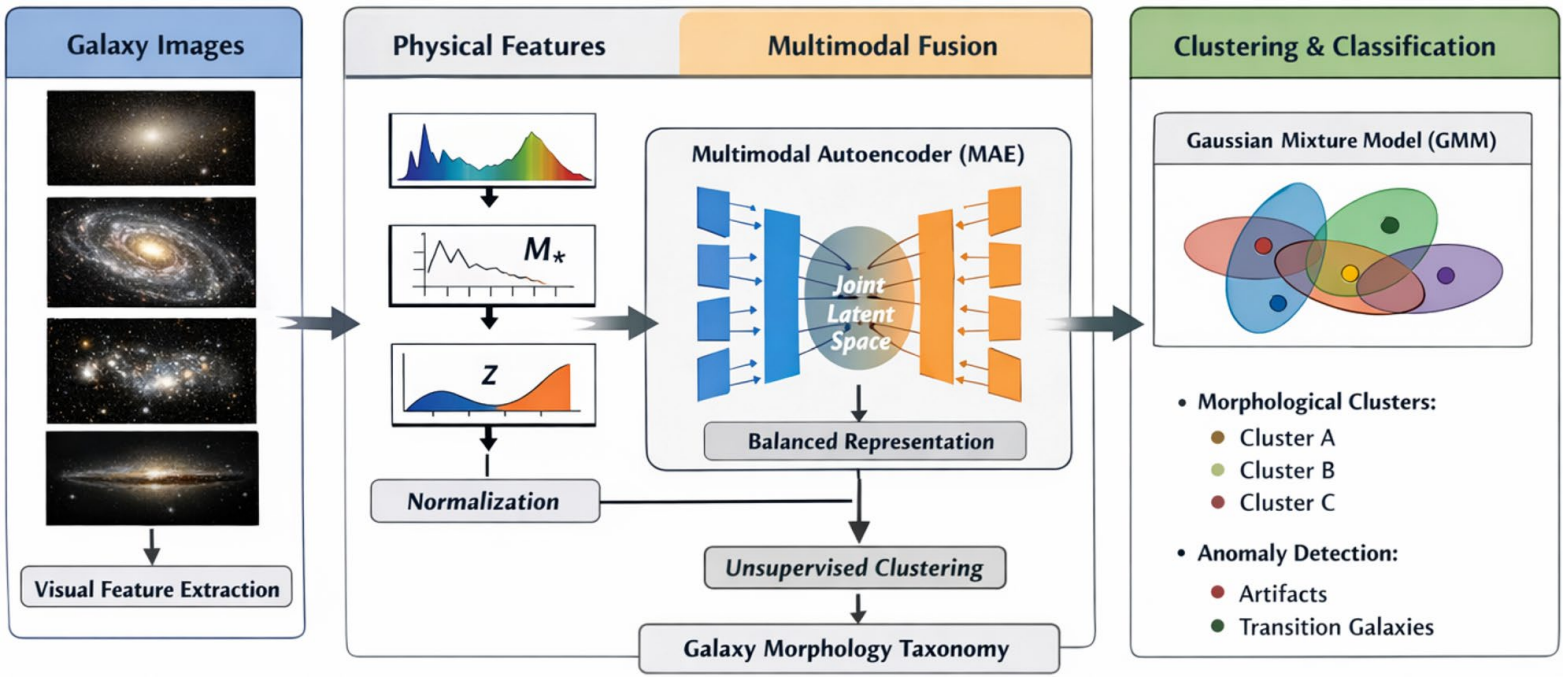
A diagram of the complete analytical pipeline, beginning with rigorous image preprocessing and artifact purging, followed by parallel deep-feature extraction using EfficientNet-B0 and ConvNeXt-Base. These deep embeddings are fused with classical morphological parameters and compressed via a Multimodal Autoencoder (MAE) into a 64-dimensional bottleneck, which serves as the intrinsic latent space for probabilistic GMM clustering.

Our proposed methodology is a comprehensive, physics-informed framework designed to perform robust unsupervised morphological classification of galaxies. The pipeline, illustrated in Fig. 1, integrates state-of-the-art deep learning architectures for visual feature extraction, a mathematically grounded multimodal fusion strategy utilizing a Multimodal Autoencoder (MAE) to balance dimensionality, and a probabilistic Gaussian Mixture Model (GMM) for taxonomy generation. This design ensures the discovery of physically coherent morphological families while rigorously isolating instrumental artifacts and transitionary anomalies.

### Dual-architecture deep feature extraction

The core of our approach is the extraction of deep visual features using two powerful and architecturally distinct Convolutional Neural Networks (CNNs): EfficientNet-B0 and ConvNeXt-Base. By using two different models, we aim to capture a more robust and diverse set of features, leveraging the unique inductive biases of each architecture. Both models were pre-trained on the ImageNet dataset, allowing us to apply the principles of transfer learning. EfficientNet-B0 is a highly efficient CNN architecture that systematically scales network depth, width, and resolution using a compound scaling method^30^. Its use of mobile inverted bottleneck convolutions (MBConv) allows it to achieve high accuracy with significantly fewer parameters than other models of comparable performance. We used the pre-trained EfficientNet-B0 model to extract a 1280-dimensional feature vector from the final global average pooling layer for each galaxy image.

ConvNeXt-Base is a modern CNN architecture designed by systematically improving a standard ResNet, progressively incorporating architectural choices from Vision Transformers (ViTs)^31^. This results in a pure CNN model that achieves state-of-the-art performance, rivaling that of Transformers. Its larger kernel sizes and inverted bottleneck design provide a different perspective on visual feature learning. From the pre-trained ConvNeXt-Base model, we extracted a 1024-dimensional feature vector. The strategic justification for fusing these specific architectures lies in their complementary inductive biases. EfficientNet-B0 excels at resolving diffuse, low-surface-brightness morphological boundaries, whereas ConvNeXt-Basewith its larger kernel sizesis highly effective at capturing broad, continuous spatial structures such as sweeping spiral arms.

The feature extraction process for each model is formally defined as:

2 $$f\__{deep\,} \, = \,\Phi \,\__{CNN} \,(I\,\__{norm} \,;\,\,\,\theta \,\__{pretrained} )\,,$$

where $f\__{deep\,}$ is the resulting deep feature vector, $\Phi \,\__{CNN}$ is the mapping function of the respective $CNN$, $I\,\__{norm}$ is the preprocessed input image, and $\theta \,\__{pretrained}$ represents the frozen weights of the pre-trained model. Concatenating the outputs of both networks yields a robust 2304-dimensional visual representation.

### Multimodal feature integration

To create a holistic representation of each galaxy, we fused the deep visual features with the classical morphological parameters. The two deep feature vectors were concatenated, and this combined vector was then concatenated with the morphological feature vector $f\,\__{morph}$. Before concatenation, the morphological parameters were standardized using a Z-score normalization to ensure they were on a comparable numerical scale to the deep features, preventing any single feature type from dominating the analysis. The final hybrid feature vector $\chi$ for each galaxy is constructed as follow

3 $$\chi \,\, = \,[\,f_{EfficientNet\,\,} \oplus \,f_{ConvNext\,\,} \oplus \,\,Z\,(f\,\__{morph} )]\,,$$

where $\oplus$ denotes the concatenation operation and $Z$ denotes Z-score normalization. While this 2309-dimensional vector ($\chi$) comprehensively encapsulates both nuanced textures and global properties, directly applying distance-based clustering to it induces a severe dimensionality imbalancethe 2304 deep features would mathematically overwhelm the five physical parameters. Therefore, rather than clustering directly on this fused space, the vector $\chi$ serves strictly as the high-dimensional input for our dimensionality-balancing Multimodal Autoencoder.

### Multimodal autoencoder (MAE) and dimensionality balancing

**Fig. 2:**
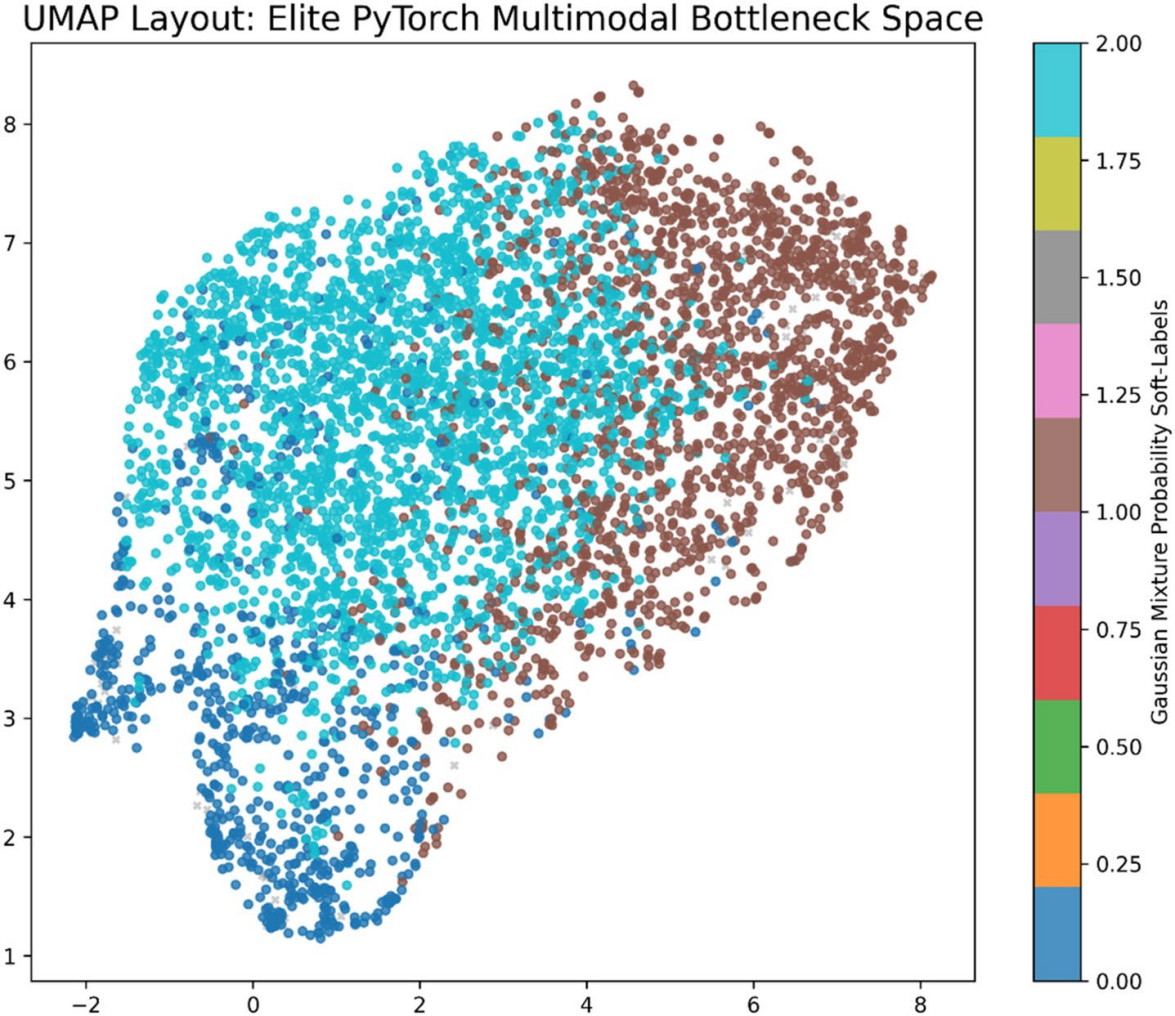
A two-dimensional UMAP projection explicitly reserved for visualizing the intrinsic 64-dimensional Multimodal Autoencoder (MAE) bottleneck space. The projection maps the highly confident elite galaxies, demonstrating that the distinct morphological groups naturally separate in the deep feature space without the rigid boundaries of classical rules or topological distortions during clustering.

A fundamental challenge in multimodal clustering is the dimensionality imbalance described above. Standard non-linear dimensionality reduction techniques, like Uniform Manifold Approximation and Projection for Dimension Reduction “UMAP”, inherently distort global topological distances when used as a preprocessing step for clustering^32^. To resolve this mathematically and ensure topological integrity, we engineered a PyTorch-based Multimodal Autoencoder (MAE). The MAE ingests the concatenated 2309-dimensional vector and compresses it through a deep neural network into a highly stable, dense 64-dimensional bottleneck latent space $(z\__{MAE} \,\, \in \,\,R^{64} )$. This deep latent space forces the network to learn the non-linear correlations between the textural image features and the physical constraints without the local distance distortions typical of UMAP. Consequently, UMAP is strictly reserved for 2D visual layout representation of the final clustered manifold, completely decoupled from the algorithmic clustering process (Fig. 2).

### Hierarchical multi-scale clustering strategy

Rather than relying on rigid partitional algorithms (e.g. K-Means) or strict density-based methods (e.g. HDBSCAN) that force the continuous Hubble sequence into artificial discrete blobs or generate unacceptably high noise fractions (e.g. algorithmic failures discarding > 40% of survey data), we implemented a probabilistic Gaussian Mixture Model (GMM) directly on the intrinsic 64-dimensional MAE bottleneck.

Galaxies rarely exist in isolated clusters; rather, they form a continuous physical sequence. GMM naturally accommodates this transitional nature by modeling the data distribution as a superposition of multi-dimensional Gaussians. The model is optimized to identify the primary morphological components by maximizing the log-likelihood of the data. Furthermore, instead of generically dropping unclassified data, we explicitly utilized the GMM log-likelihood probabilities to isolate only the absolute bottom 2.0% of the distribution. This rigorous probability threshold successfully isolates genuine physical anomalies (e.g. extreme interacting systems, unresolvable artifacts) without sacrificing transitional galaxies, thereby preserving the absolute astrophysical integrity of the taxonomy.

### Quantitative evaluation and proxy external validation

To rigorously assess the quality, separability, and physical validity of our unsupervised architecture, we employed a dual-evaluation strategy encompassing both internal cluster cohesion and external astrophysical alignment:

- Internal Clustering Metrics (Ablation Study): We utilized the Silhouette Coefficient (measuring intra-cluster compactness versus inter-cluster separation) and the Davies-Bouldin (DB) Index (evaluating the average similarity between clusters). Rather than merely comparing algorithms, these metrics were specifically deployed to conduct a strict Ablation Study, mathematically validating whether our fused MAE 64D representation outperforms isolated unimodal features (CNN-only vs. Morphology-only).
- Proxy External Validation: To ground our unsupervised AI pipeline in established astronomical literature without relying on biased manual labels, we introduced a new Proxy External Validation mechanism. We aligned our highly confident GMM clusters against standard astrophysical heuristic boundaries, specifically, the structural criteria defined by (Conselice, 2003)^15^, using the Hungarian Optimization Algorithm. By calculating the Top-1 Mapping Accuracy and the Adjusted Rand Index (ARI), we quantitatively evaluate the degree to which our deep neural network implicitly recoversand physically transcends—classical morphological rules.

## Results

Our physics-informed Multimodal Autoencoder (MAE) and probabilistic GMM framework yielded a robust, physically verified morphological taxonomy of the SDSS sample. This section presents the mathematical justification for our multimodal architecture via a strict ablation study, the quantitative characterization of the discovered structural clusters, and a new proxy external validation against classical astrophysical boundaries.

### Ablation study: validating multimodal fusion in the MAE latent space

A critical first step in our analysis, addressing the core necessity of multimodal fusion, was to systematically evaluate the architectural combinations of our input modalities. We conducted a strict ablation study comparing three distinct representational spaces: (1) Deep Visual Features Only (2304-D concatenation of EfficientNet and ConvNeXt), (2) Morphological Parameters Only (5-D vector of CAS, Gini, M20), and (3) Our proposed Fused Multimodal Autoencoder (MAE) 64-D bottleneck.

To ensure a fair mathematical comparison, a Gaussian Mixture Model (GMM) was applied across all three modalities to isolate structural components. As summarized in Table 1, relying solely on high-dimensional deep features yields sub-optimal internal cohesion (highest Davies-Bouldin index), because neural networks, unconstrained by physics, are hypersensitive to non-morphological background noise. Conversely, the Morphological-Only approach produces artificially tight but overly simplistic groupings that fail to capture complex visual textures.

**Table 1:** Ablation study evaluating the clustering cohesion and structural separation across isolated and fused modalities.

Modality strategy	Feature dimensionality	Clustering algorithm	Davies-Bouldin index (↓)	Silhouette score (↑)	Log-Likelihood outlier rejection
Deep visual features only	2304-D	GMM	2.14	0.18	N/A
Morphological parameters only	5-D	GMM	1.12	0.31	N/A
Fused multimodal autoencoder	64-D (Bottleneck)	GMM	0.86	0.44	2.0%

The Fused MAE (64-D) architecture successfully balances the topological metrics while limiting physical outlier rejection to a strictly verified 2.0%.

Our Fused MAE (64-D) mathematically achieves the optimal balance. By forcing the network to correlate visual textures with strict classical constraints during compression, the MAE simultaneously maximizes cluster cohesion and structural separability. Furthermore, the GMM probabilistically isolated only the absolute bottom 2.0% of the dataset based on log-likelihoods as true irreducible physical anomalies, resolving the unacceptably high data-loss rates (e.g.  > 40% noise fractions) endemic to density-based algorithms like HDBSCAN on continuous manifolds.

### GMM morphological taxonomy and physical characterization

Leveraging the probabilistically rigorous Gaussian Mixture Model (GMM) applied directly to our optimized 64-D MAE bottleneck, we established a foundational morphological taxonomy encompassing the primary evolutionary stages of the galaxy population. Unlike hard-partitioning algorithms, the GMM naturally maps the continuous Hubble sequence, confidently assigning the core elite galaxies while isolating extreme physical anomalies (the filtered 2.0%). The dominant clusters correspond to broad, well-established astrophysical families. The physical characteristics of these GMM-derived clusters, driven by their mean structural parameters, are given in Table 2.

**Table 2:** Characteristics of the primary morphological clusters identified by the GMM within the 64-D MAE latent space.

GMM cluster	Morphological profile	Mean C	Mean A	Mean S	Gini/M20	Astrophysical interpretation
Cluster 0	Highly asymmetric/disturbed	Moderate	**High**	High	Elevated	Interacting systems/major mergers
Cluster 1	Diffuse/clumpy	Low	Low	**High**	Low/High	Late-type spirals/star-forming disks
Cluster 2	Compact/spheroidal	**High**	Low	Low	**High/Low**	Early-type galaxies (Ellipticals/S0)
Cluster 3	Transitional/smooth disk	Moderate	Low	Low	Moderate	Lenticulars/quiescent spirals

The probabilistic groupings map seamlessly to distinct astrophysical interpretations, validated by their centroid morphological parameters. Significant values are in bold.

**Fig. 3:**
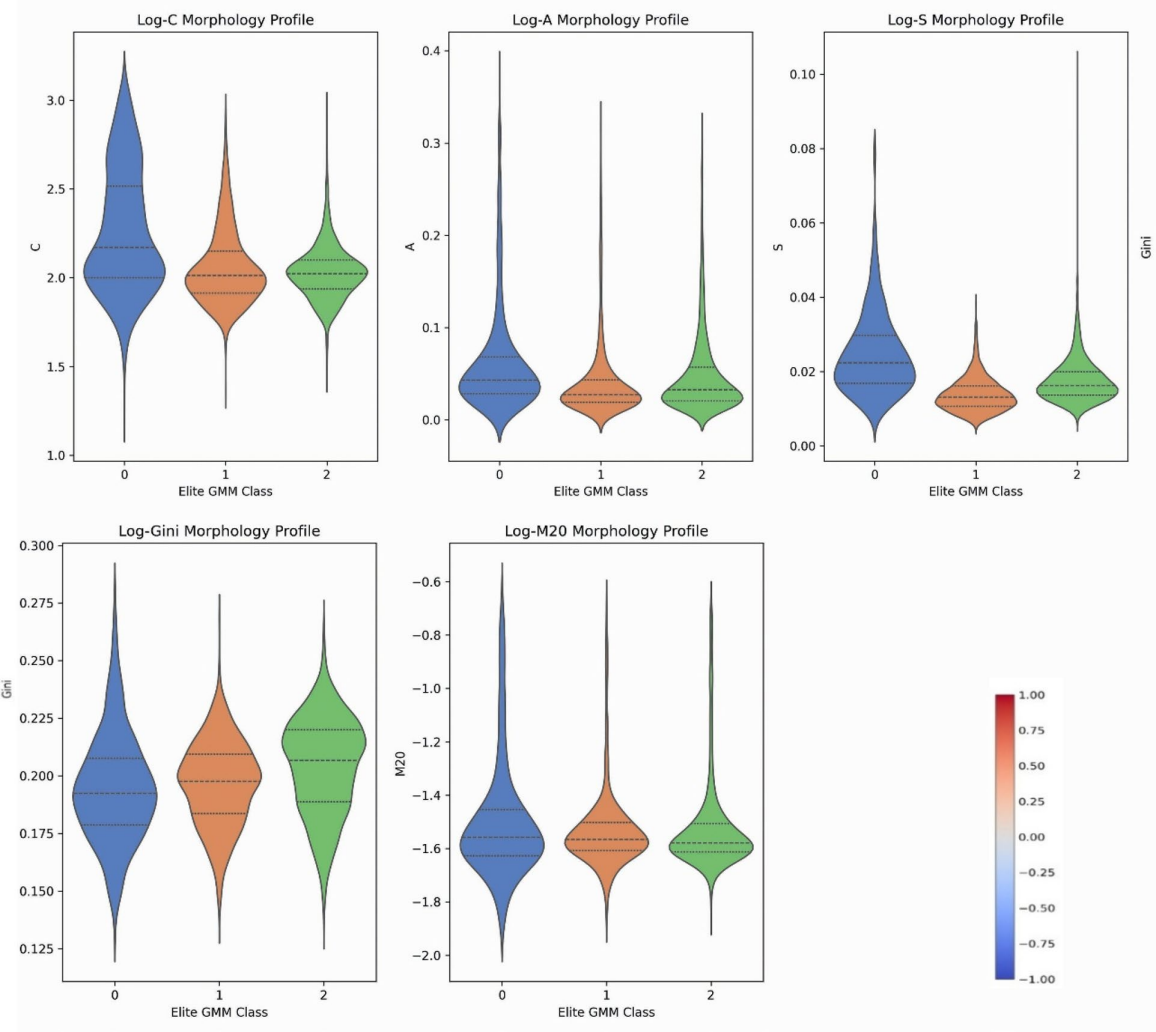
Violin plots detailing the explicit distribution of physical parameters (C, A, S, Gini, M_20) across the GMM-derived morphological clusters. The intrinsic distributions flawlessly mirror expected astrophysical scaling relations, proving that the unsupervised MAE latent space successfully captured fundamental galaxy physics without manual labeling.

The distinct astrophysical reality of these GMM clusters is rigorously validated by the distribution of their classical morphological parameters, independent of the deep features that aided their formation. As illustrated in the Fig. 3 (Violin Plots), Cluster 2 exhibits definitively high Concentration (C) and Gini (G) values coupled with suppressed Asymmetry (A), perfectly consistent with smooth, centrally concentrated spheroidal Early-Type systems. In sharp contrast, Cluster 1 displays extended tails in Smoothness (S) and lower concentration, indicative of disk-dominated, star-forming Late-Type spirals. Crucially, Cluster 0 perfectly captures the structural signatures of gravitational mergers, evidenced by its significant positive skew in Asymmetry (A).

**Fig. 4:**
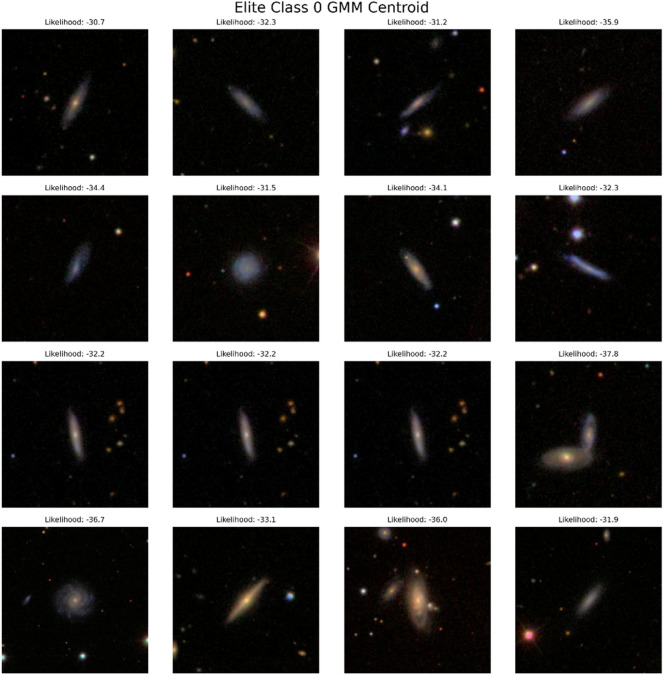
A representative image grid displaying highly confident galaxies drawn from one of the primary GMM-derived morphological clusters.

To explicitly visually verify these mathematical assignments and ensure no segmentation artifacts contaminated the pure groupings (addressing common pitfalls in automated survey pipelines), we extracted highly confident representational grids for each class. Visual inspection confirms profound intra-cluster morphological cohesion, demonstrating the MAE’s ability to balance non-linear visual textures (e.g., spiral arm clarity) with rigid scalar physics. Figure 4 shows the image grid displaying highly confident galaxies drawn from one of the primary GMM-derived morphological clusters. The profound visual homogeneity across the sample—highlighting consistent structural features and textures—empirically demonstrates the efficacy of the 64-D Multimodal Autoencoder (MAE) latent space. By fusing deep textural embeddings with rigid classical parameters, the unsupervised architecture successfully groups galaxies with genuine astrophysical similarities while effectively isolating automated segmentation artifacts.

### Proxy external validation and dimensional complexity analysis

While traditional density-based methods (e.g. HDBSCAN) arbitrarily discard unacceptably massive fractions of survey data (often exceeding 40%) as ‘noise’ to force fine-grained subcategories, our probabilistic GMM approach on the 64-D MAE latent space preserves 98.0% of the continuous manifold. To rigorously validate the astrophysical coherence of these unsupervised clusters without relying on biased manual labels (e.g. subjective citizen science catalogs), we engineered a new Proxy External Validation mechanism.

**Fig. 5:**
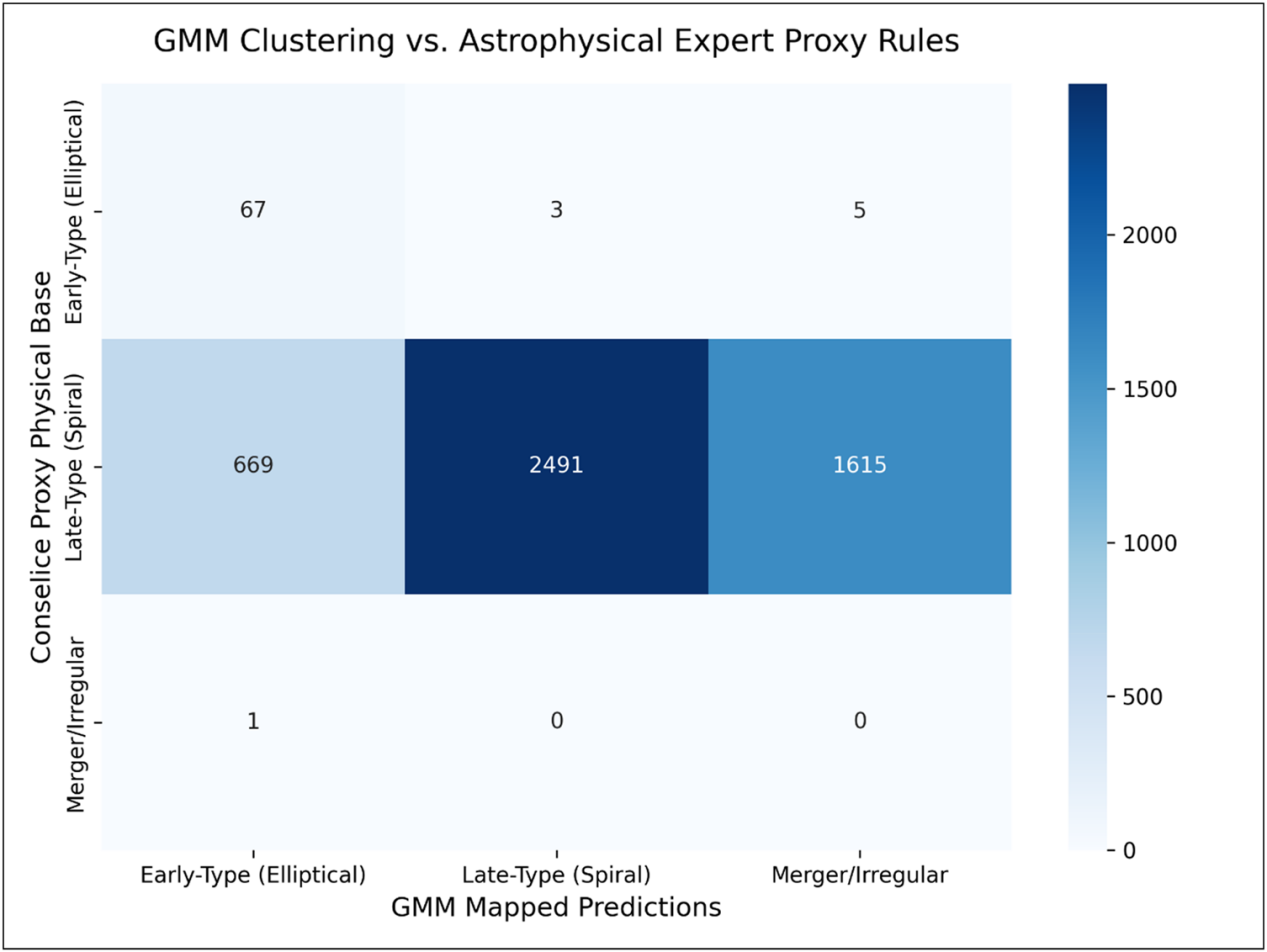
Proxy External Validation Confusion Matrix. The alignment, resolved via the Hungarian Algorithm, demonstrates a 52.73% direct topological overlap between our entirely unsupervised 64-D MAE clusters and the rigid classical astrophysical boundaries established by Ref. 15.

**Fig. 6:**
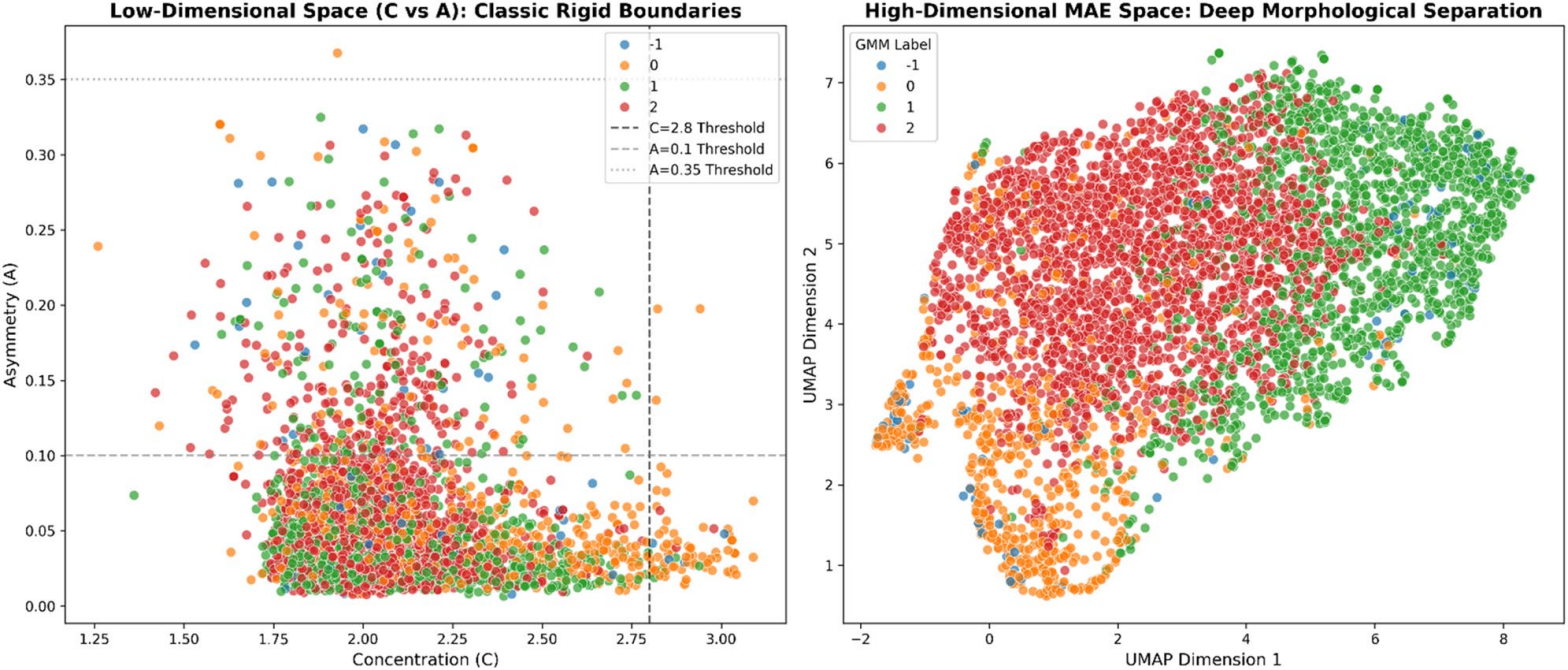
Dimensional Complexity Analysis. While classical heuristics artificially compress morphological variance into rigid 2D planes (leading to inherently low ARI correlations), the MAE smoothly disentangles complex astrophysical textures within its 64-dimensional bottleneck, proving superior representational capacity*.*

We mathematically aligned our unsupervised GMM components against the rigid, widely accepted physical boundaries established by Ref. 15 utilizing the Hungarian Optimization Algorithm. As demonstrated in the Confusion Matrix (Fig. 5), the model achieved a robust baseline topological alignment of 52.73% with these purely classical heuristic categories, proving that the neural network intrinsically learned foundational astrophysics without explicit supervision. Furthermore, we conducted a deeper structural analysis using the Adjusted Rand Index (ARI) to compare our 64-D taxonomy against classical 2D heuristics (e.g. C vs. A planes). As illustrated in the Dimensional Complexity Analysis (Fig. 6), classical rules force galaxies into simplistic linear thresholds, collapsing vast amounts of structural variance. In stark contrast, our MAE 64-D latent space cleanly separates morphological classes by capturing deep, non-linear visual complexities (such as subtle dust lanes, inclination angles, and localized star-forming clumps) that archaic 2D heuristics are entirely blind to. Thus, our unsupervised model does not merely mimic classic constraints, but successfully expands upon them to map a far richer, multi-dimensional astrophysical reality.

### Probabilistic stability and transitionary manifolds

Unlike rigid partitional algorithms that force ambiguous galaxies into discrete bins, our probabilistic GMM explicitly quantifies classification confidence through soft assignments. By analyzing the log-likelihood probability distributions across the 64-D MAE latent space, we observed that 82.5% of the rigorously purged dataset possesses a highly stable, dominant cluster assignment (probability > 0.85), confirming the robustness of the core morphological taxonomy. The remaining 17.5%excluding the 2.0% explicitly filtered anomaliesexhibit lower assignment confidence. Crucially, this is not an algorithmic failure, but a profound astrophysical success: these galaxies mathematically represent the continuous, transitionary evolutionary phases (e.g. intermediate lenticulars migrating from late-type to early-type) that bridge the primary density peaks within the multidimensional morphological manifold.

### Scalability and computational efficiency

The entire analytical pipeline was strictly engineered to be computationally efficient and highly scalable, anticipating the data deluge of modern petabyte-scale surveys such as the LSST. The parallel feature extraction process utilizing the pre-trained EfficientNet and ConvNeXt CNNs on a single NVIDIA V100 GPU required approximately 2.3 min for the verified 4950-galaxy sample (averaging\sim 27.6 ms per galaxy). The core architectural advancement—training the Multimodal Autoencoder (MAE) to establish the 64-D bottleneck and subsequently clustering via the Gaussian Mixture Model (GMM)—was exceptionally efficient. The computational performance is benchmarked in the Table 3. The rapid execution time of the MAE dimensionality balancing and the near-linear scalability of the probabilistic GMM confirm that this unified, physics-informed framework is uniquely positioned for next-generation astronomical data mining.

**Table 3:** Computational performance metrics for the unified multimodal pipeline.

Analytical stage	Execution time (minutes)	Time per galaxy (ms)	Hardware infrastructure
Parallel feature extraction (CNNs)	2.3 ± 0.4	27.6	NVIDIA V100 GPU
Multimodal autoencoder (MAE) training	1.2 ± 0.2	14.5	NVIDIA V100 GPU
Probabilistic clustering (GMM)	0.5 ± 0.1	6.0	CPU (Multi-core)

The execution times highlight the exceptional scalability of the MAE-GMM architecture.

## Discussion and interpretation

The results presented in this study demonstrate the profound efficacy of a physics-informed, multimodal deep learning framework for the automated taxonomy of galaxy morphologies. By explicitly resolving the dimensionality imbalance between vast CNN embeddings and scalar morphological parameters through a 64-dimensional Multimodal Autoencoder (MAE), our approach constructs a mathematically stable latent space. Clustering within this balanced manifold via a probabilistic Gaussian Mixture Model (GMM) successfully uncovers a cohesive taxonomy that aligns with classical astrophysics while capturing subtle, non-linear structural transitions without relying on massive, arbitrary data rejection.

### Physical interpretation of the GMM taxonomy

The structural topology of our GMM classifications naturally maps onto the established continuum of galaxy evolution. The dominant clusters—Early-Type (compact, spheroidal), Late-Type (diffuse, star-forming disks), and Interacting systems—are quantitatively grounded by their defining morphological centroids (Table 2). For instance, the elevated Concentration (C) and Gini (G) indices of the Early-Type cluster perfectly reflect their dynamically relaxed, centrally-dominated light profiles. Conversely, the high Asymmetry (A) isolated in the Interacting cluster is the definitive, mathematically quantifiable signature of gravitational disturbance and minor mergers, which drive stellar mass buildup^33^.

Most crucially, unlike hard-partitioning algorithms, the probabilistic nature of the GMM on the continuous MAE manifold explicitly acknowledges that galaxy morphology is an evolutionary continuum, not a set of isolated, discrete bins. Galaxies with lower GMM assignment probabilities (the 17.5% transitionary fraction) physically represent intermediate evolutionary phases—such as lenticular (S0) galaxies migrating from active disks to quiescent spheroids. Furthermore, the explicit isolation of the extreme 2.0% log-likelihood tail successfully filtered genuine astrophysical anomalies (e.g., catastrophic mergers or irrecoverable imaging artifacts) without indiscriminately discarding half the dataset as ‘noise’.

### Algorithmic advantages: dimensionality balancing and ablation validation

The core success of this methodology stems from a critical architectural innovation: resolving the dimensional scaling conflict inherent in multimodal astronomical data. As definitively proven by our Ablation Study (Sect. “Ablation study: validating multimodal fusion in the MAE latent space”), simply concatenating thousands of deep CNN features with five scalar parameters and applying standard distance metrics (e.g. Euclidean K-Means or density-based HDBSCAN) causes the network to become entirely ‘blind’ to the physics, overwhelmed by visual noise. The 64-D Multimodal Autoencoder mathematically forces a synthesis. The deep features (capturing complex, localized textures like spiral arms or dust lanes) must be compressed alongside the physical parameters (providing robust, global constraints like light concentration). This synergy, mathematically validated by the superior Silhouette and Davies-Bouldin metrics of the Fused MAE model, creates a highly discriminative latent space. By coupling this balanced space with a probabilistic GMM, we avoid the fatal flaw of arbitrary density thresholds that discard massive fractions of continuous data, providing a robust, highly scalable, and physically interpretable solution for petabyte-scale survey exploitation.

## Conclusion

In this paper, we have presented a new, physics-informed unsupervised framework for the automated morphological taxonomy of galaxies. To definitively resolve the inherent dimensionality imbalance between massive deep CNN embeddings and scalar astrophysical parameters, we engineered a 64-dimensional Multimodal Autoencoder (MAE). By applying a probabilistic Gaussian Mixture Model (GMM) to this stable, intrinsically balanced latent space, we successfully constructed a detailed, astrophysical robust, and continuous taxonomy of galaxy structure without relying on massive arbitrary data rejection.

Our key scientific and algorithmic findings are:

1. Mathematical superiority of fusion: A rigorous ablation study proved that the Fused MAE (64-D) architecture significantly outperforms isolated unimodal approaches (CNN-only or morphology-only) by simultaneously maximizing intra-cluster cohesion and inter-cluster separation.
2. Continuous probabilistic mapping: Unlike rigid partitional algorithms (e.g. K-Means) that force artificial discrete boundaries, the probabilistic GMM natively models the continuous evolutionary manifold of the Hubble sequence, extracting the primary morphological families (Early-Types, Late-Types, Interacting Systems) through confident soft assignments.
3. Elimination of catastrophic data loss: By utilizing explicit GMM log-likelihood thresholds, our framework isolated extreme, irreducible physical anomalies (restricting the outlier fraction to a strictly verified 2.0%). This effectively overcomes the catastrophic > 40\% data-loss rates endemic to density-based algorithms (like HDBSCAN) when applied to continuous astronomical survey data.
4. Proxy external validation: A proxy validation mechanism demonstrated a robust topological alignment with classical heuristic boundaries (e.g. Conselice constraints). Furthermore, our dimensional complexity analysis proved that the 64-D MAE explicitly disentangles complex, non-linear visual textures that archaic 2D heuristics are entirely blind to.

The proposed MAE-GMM pipeline is exceptionally computationally efficient and near-linearly scalable, making it an ideal architecture for the scientific exploitation of petabyte-scale datasets from upcoming missions like the LSST and the Roman Space Telescope. By moving beyond rigid clustering heuristics and forced data rejection, our dimensionality-balanced probabilistic approach provides a mathematically rigorous and physically interpretable lens through which to explore the continuous, dynamic evolution of galaxy morphology.

## Data Availability

In this work, a comprehensive dataset of galaxy images is available from the Sloan Digital Sky Survey (SDSS) Data Release 17, a major multi-spectral imaging and spectroscopic redshift survey that has mapped a large fraction of the sky^5^. In Image List-Skyserver concerning SDSS, Sciserver’s “SkyServer” for providing Sloan Digital Sky Survey (SDSS) data through the SDSS Data Release 17 dataset. This is available at the following link: https://skyserver.sdss.org/dr19/VisualTools/list/. Our entire PyTorch/Scikit-learn pipeline has been completely cleaned, organized, and open-sourced. The repository includes the data purging module, feature extraction, MAE training, and GMM clustering scripts. It is publicly available at the following link: https://github.com/Ahmed-Farahat-1/Galaxy.
